# Hierarchical TiO_2_ Layers Prepared by Plasma Jets

**DOI:** 10.3390/nano11123254

**Published:** 2021-11-30

**Authors:** Radek Zouzelka, Jiri Olejnicek, Petra Ksirova, Zdenek Hubicka, Jan Duchon, Ivana Martiniakova, Barbora Muzikova, Martin Mergl, Martin Kalbac, Libor Brabec, Milan Kocirik, Monika Remzova, Eva Vaneckova, Jiri Rathousky

**Affiliations:** 1 J. Heyrovsky Institute of Physical Chemistry, Czech Academy of Sciences, Dolejskova 3, 182 23 Prague, Czech Republic; ivana.martiniakova@jh-inst.cas.cz (I.M.); barbora.muzikova@jh-inst.cas.cz (B.M.); martin.mergl@jh-inst.cas.cz (M.M.); martin.kalbac@jh-inst.cas.cz (M.K.); libor.brabec@jh-inst.cas.cz (L.B.); milan.kocirik@jh-inst.cas.cz (M.K.); monika.remzova@jh-inst.cas.cz (M.R.); eva.vaneckova@jh-inst.cas.cz (E.V.); 2 Institute of Physics, Czech Academy of Sciences, Na Slovance 2, 182 21 Prague, Czech Republic; olejn@fzu.cz (J.O.); pnovotna@fzu.cz (P.K.); hubicka@fzu.cz (Z.H.); duchon@fzu.cz (J.D.)

**Keywords:** TiO_2_, P25, photocatalysis, NO_x_ and phenol abatement, external quantum efficiency

## Abstract

Heterogeneous photocatalysis of TiO_2_ is one of the most efficient advanced oxidation processes for water and air purification. Here, we prepared hierarchical TiO_2_ layers (Spikelets) by hollow-cathode discharge sputtering and tested their photocatalytic performance in the abatement of inorganic (NO, NO_2_) and organic (4-chlorophenol) pollutant dispersed in air and water, respectively. The structural-textural properties of the photocatalysts were determined via variety of physico-chemical techniques (XRD, Raman spectroscopy, SEM, FE-SEM. DF-TEM, EDAX and DC measurements). The photocatalysis was carried out under conditions similar to real environment conditions. Although the abatement of NO and NO_2_ was comparable with that of industrial benchmark Aeroxide^®^ TiO_2_ P25, the formation of harmful nitrous acid (HONO) product on the Spikelet TiO_2_ layers was suppressed. Similarly, in the decontamination of water by organics, the mineralization of 4-chlorophenol on Spikelet layers was interestingly the same, although their reaction rate constant was three-times lower. The possible explanation may be the more than half-magnitude order higher external quantum efficacy (EQE) compared to that of the reference TiO_2_ P25 layer. Therefore, such favorable kinetics and reaction selectivity, together with feasible scale-up, make the hierarchical TiO_2_ layers very promising photocatalyst which can be used for environmental remediation.

## 1. Introduction

The fabrication of hierarchical TiO_2_-based photocatalysts at the micro/nanometer scales has been identified as a promising strategy for environmental application. Properly designed hierarchical structures, including their porosity and morphology, can not only enhance light harvesting and improve molecular diffusion/transport kinetics, but also increase the surface area and the concentration of active sites, which accelerates the surface reaction rate. Additionally, the conduction and valence bands of hierarchically assembled nanomaterials can be adjusted by reducing the size of building blocks to the nanoscale level and taking advantage of the quantum size effect.

Recently, new methods for effective deposition of TiO_2_ nanostructured thin layers have been developed. The layers should exhibit a high crystallinity for effective transport of photogenerated carries to the surface, optimal structure, and a large surface area. Simultaneously, the adhesion and cohesion of the hierarchical nanomaterial onto the substrate should be very high. If not, there is the risk of photocatalyst deterioration.

As a possible preparation technique, a plasmatic deposition including reactive sputtering or evaporation of metallic materials in oxygen gas can be used. A typical example is a reactive magnetron sputtering system of titanium sputtered in Ar and O_2_ gas mixture atmosphere [1,2,3]. This method can provide TiO_2_ layers with excellent adhesion and semiconductor properties, fulfilling sufficient photocatalytic performance as well [4,5]. Furthermore, the reactive magnetron sputtering of TiO_2_ is routinely used at the industrial scale to coat a large surface (i.e., windows or foils). On the other hand, these layers are usually perfectly flat and dense, and the surface area of such a photocatalyst is smaller in comparison with, for example, sol-gel preparation method [6].

The above-mentioned technique has been used in [7]. The authors prepared manocrystalline TiO_2_ with a hierarchical microstructure by PLD (plasmatic layer deposition). Superior photocatalytic parameters were observed for these TiO_2_ nanostructures in comparison with P25 powders. On the other hand, the hollow cathode plasma jet deposition technique presented in the paper offers the possibility of depositing these TiO_2_ nanostructures on large areas with a high deposition rate and the process can be easily implemented in industrial deposition machines.

The deposition rate of reactive magnetron sputtering has been optimized by new configurations of reactive gas flow controls but is still not high enough to solve all the problems of deposition process effectivity. In recent years, alternative plasmatic methods employing reactive sputtering were developed. The system with RF or DC hollow cathode discharges and flowing plasma were applied for the fast deposition of various oxide layers [8,9,10]. This method provided good-quality deposited layers, a large effective surface and a high deposition rate [9]. Several configurations with parallel plate hollow cathode and plasma flows were developed and applied for effective oxide deposition on a large area [11]. Some modifications of plasma systems with DC hollow cathode discharge sputtering and flowing plasma were investigated as an effective source of metallic and metal oxide clusters [12,13]. These clusters are formed inside the hollow cathode region in plasma flow or at the outlet of this cathode due the high pressure in that region and the higher probability of the three particle collision process and the cluster formation directly in the gas phase [14].

The further modification with the hot DC hollow cathode discharge and the flow of high-density plasma was developed in a multi-plasma jet version and applied for the deposition of TiO_2_ and Co_3_O_4_ thin layers with a high deposition rate on large areas [15,16]. These oxide layers exhibit good adhesion and large specific area of the surface and high roughness of the surface.

From an application point of view, the feasibility of up-scaling is of the utmost importance. The presented system uses four independent hollow cathode discharges with plasma jets in line but in principle, more independent hollow cathode discharges in line can be arranged in this way and a longer tape can be coated when the substrate is stationary. For coating of large areas, the substrate linear movement below this line of plasma jets can be implemented and uniform thin film deposition can be achieved in this way.

This paper describes the application of this modified multi-plasma jet system with DC hollow cathodes for the deposition of TiO_2_ thin layers with a specific microstructure and surface roughness. These TiO_2_ layers were investigated for photocatalytic applications. These prepared hierarchical TiO_2_ layers were subject to a detailed characterization of various features of their hierarchical structure and to measurements of their electrical properties. Moreover, the activity and selectivity of the layers was tested in the photocatalytic oxidation of NO_x_ species in the gas phase, and investigated for photocatalytic applications. In addition to the conversion of primary pollutants (i.e., NO and NO_2_ in the present study), the photocatalytic tests provided the data on formation of potentially toxic products or intermediates. This issue if of major importance and should not be overlooked. Alternatively, the performance of hierarchical layers was tested in the degradation of 4-chlorophenol, exhibiting a high degree of beneficial mineralization.

## 2. Materials and Methods

### 2.1. TiO_2_ Layer Preparation

#### Spikelet Layers Preparation via Hollow-Cathode Discharge (HCD) Sputtering

**Figure 1:**
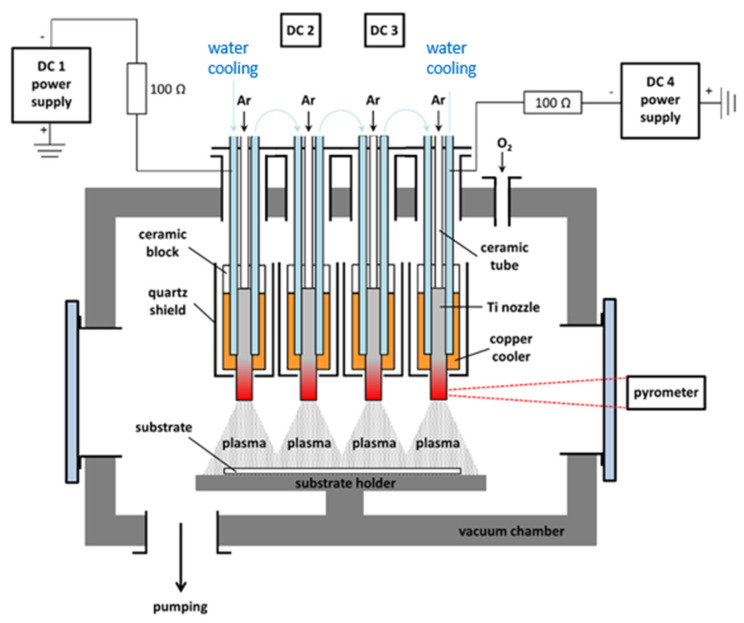
Scheme of multi-plasma-jet system with four independent DC hollow cathodes used for deposition of hierarchical TiO_2_ Spikelet layers.

TiO_2_ thin layers were deposited by multi-plasma-jet system with four independent titanium DC hollow cathodes. A similar system with cobalt or tungsten cathodes was recently used for the preparation of Co_3_O_4_ [16] or WO_3_ material [17] and was already described in detail in ref. [15]. It enables extremely fast reactive sputtering of oxide layers. The basic scheme of the system is depicted in Figure 1. It contains four independent titanium hollow cathodes arranged linearly in a row. The distance between the centers of two adjacent cylindrical Ti nozzles is 36 mm. The internal diameter and the length of the nozzle is 5 and 48 mm, respectively. Each nozzle is embedded inside with a water-cooled copper cooler, the bottom part of each nozzle being located outside the cooler. Therefore, this part of nozzle is extremely heated during the deposition. Its temperature can reach up to 1600 °C depending on the discharge parameters. This principle makes it feasible to combine sputtering of the titanium atoms with their thermal evaporation, thus greatly increasing the deposition rate.

Each nozzle was connected via a 100-ohm resistor to its own DC power supply. This resistor acts as a discharge stabilizer to help prevent unwanted discharge oscillations or arc ignition. Hollow cathode discharge was operated in current control mode with a stable discharge current 1.5 A per nozzle. Under such conditions, the typical absorbed power per each nozzle was 600 W; a typical hollow cathode temperature was about 1300 °C. The entire deposition system was placed inside the vacuum chamber, and prior deposition, this chamber is pumped down by a combination of turbo-molecular, roots and rotary pumps to a base pressure of 10^−3^ Pa. Working gases (Ar and O_2_) enter the chamber through two different inlets. While the inert gas (Ar-99.996% purity) was supplied directly through the titanium nozzles, the reactive gas (O_2_-99.999% purity) was supplied to the chamber by a separate inlet. Thanks to this, no oxidation of the titanium nozzles during deposition could occur, so the discharge burned all the time in metallic mode only.

During the deposition, the gas pressure in the reactor was maintained at 15 Pa. However, the pressure inside the hollow cathode was significantly higher for the reasons described in [18]. Considering that the speed of the gas at the nozzle outlet was sonic, the minimum pressure inside the cathode was in the range of 120–200 Pa. The flow of argon through each nozzle was kept at 150 cm^3^·min^−1^ (i.e., 600 cm^3^·min^−1^ of Ar in total); the oxygen flow through the separate inlet was constant at 50 cm^3^·min^−1^. The distance between the nozzle outlets and substrate holder was 8 cm. The substrate temperature during deposition continuously increased from room temperature (at the beginning) up to 200 °C. The loading of the deposited Spikelet TiO_2_ was 2 mg·cm^−2^.

#### P25 Layers Preparation via Spin Coating

In order to compare the efficacy of hierarchical TiO_2_ layers, widely used commercial TiO_2_ powder P25 (Aeroxide^®^ TiO_2_ P25, Evonik, Essen, Germany) was prepared by spin-coating and tested for its photocatalytic ability. TiO_2_ was deposited in multiple layers to achieve the same weight as that of Spikelet TiO_2_ plasma jet layers (2 mg·cm^−^^2^). To increase the adhesion of a soda-lime glass, the surface of 5 × 10 cm was first abraded by Silicon Carbide of grits 220 (Top Geo, Satteldorf, Germany). Secondly, water suspension containing 29 wt% of P25 was homogenized by dispersing instrument T25 (IKA, Königswinter, Germany) for 5 min at 20,000 rpm, and afterwards, deposited on the glass plate using a spin-coater (KW-4A) at 1000 rpm. Before the photocatalytic testing, all the samples were stored in a dark chamber under nitrogen atmosphere and adjusted temperature of 25 °C.

### 2.2. TiO_2_ Layer Characterization

#### X-ray Diffraction

XRD patterns were recorded using Empyrean diffractometer in Bragg-Brentano (BB) geometry with Cu Kα radiation (λ = 0.154 nm) in 2θ range from 5° to 90° with a step size of 0.01°. The qualitative analysis was performed with a HighScore Plus 4.0 software package (PANanalytical, Malvern Panalytical, Malvern, UK).

#### Raman Spectroscopy

The Raman spectra were collected using a Ar-Kr laser at 2.41 eV. They were recorded on a LabRAM HR (Horiba Jobin-Yvon, Kyoto, Japan) Raman spectrometer interfaced with an Olympus BX microscope (objective 100× with LWD lenses to ensure a lateral resolution of 1 µm, the laser power impinging on the sample was ~1 mW to avoid heating). The spectrometer was calibrated before each series of measurements by using the F_1g_ mode of Si at 520.2 cm^−1^.

#### UV-Vis Spectroscopy

For the band-gap determinations, diffuse reflectance spectra of P25 and Spikelet layers were recorded by employing a UV/Vis/NIR spectrometer (Perkin-Elmer Lambda 1050 model, Waltham, MA, USA), equipped with a diffuse 60 mm spectralon integration sphere (2 detectors PMT/PbS). The Kubelka–Munk function (K–M) was used to determine the band gap values by performing the first derivative the K–M equation. A line is construct tangent to the point of inflection first derivative K–M function. Experimental data of diffuse reflectance were elaborated to absorption coefficient values F(R) according to the Kubelka–Munk equation: $f\left(R_{\infty }\right)={\left(1-R_{\infty }\right)}^{2}/\left(2R_{\infty }\right)$, where R is the reflectance.

#### Sorption Experiments

Sorption isotherms of Kr were measured at the boiling point of liquid nitrogen (ca. 77 K) using a 3Flex apparatus (Micromeritics, Norcross, GA, USA). The surface area was calculated by the BET equation.

#### FE-SEM, DF-TEM Coupled with EDAX

FE-SEM micrographs were collected on a Hitachi S-4800 instrument (Hitachi, Tokyo, Japan). In the magnetron, several layers of various area were deposited simultaneously: (i) onto a large-area glass (5 × 10 cm^2^) and (ii) onto silicon wafers (1 × 1 cm^2^). For SEM, the latter were used. In order to see the layer profile, the coated silicon plate was easily broken after notching the edge.

#### TEM

Transmission electron microscopy (TEM) was carried out on a FEI Tecnai TF20 X-twin microscope (FEI, Hillsboro, Oregon, USA) operated at 200 kV (FEG, 1.9 Å point resolution) with an EDAX Energy Dispersive X-ray (EDX) detector attached. Images were recorded on a Gatan CCD camera with resolution of 2048 × 2048 pixels using the Digital Micrograph software package (Gatan, Pleasanton, CA, USA). Electron diffraction patterns were evaluated using the CrysTBox software (Institute of Physics, Prague, Czech Republic) for an automated analysis of electron diffraction patterns. The lamella for TEM observation was prepared by focused-ion-beam milling in a FEI Quanta 3D.

#### DCDF-TEM

To distinguish between the two TiO_2_ phases—rutile and anatase—the Dark Field (DF) TEM technique with the Dynamic Conical Dark Field (DCDF) option was used. In the DCDF TEM technique, as well as in the conventional DF technique, the sample is illuminated by a parallel beam, and the area corresponding to the respective diffraction spot is selected in the diffraction pattern by the objective aperture. In the case of DCDF, the diffraction pattern performs a precession motion around the optical axis, and thus, diffraction spots corresponding to different orientations of the crystals gradually enter the objective aperture, i.e., the whole diffraction annulus is integrated (Figure 5b). Therefore, this technique is suitable for fine-grained materials with a random grain orientation, in which it allows to visualize all grains with a corresponding inter-planar distance within one image. The range of inter-planar distances is determined by the size of the objective aperture and the cone angle, which determines the distance of the selected annulus from the center of the diffraction pattern. During the experiment, the smallest 10-μm objective aperture was used. Due to the fact that even this smallest objective aperture did not allow a clear distinction between the diffraction rings of anatase and rutile, the following cone angle settings were used: 0.28° for anatase (green annulus) and 0.41° for the combination of anatase and rutile (red annulus). The distribution of rutile grains was obtained by subtracting the signal on the two micrographs obtained by these two settings (Figure 5d).

#### DC Measurements

An interdigital electrode (IDE) system was developed in the J. Heyrovsky Institute to collect DC characteristics of the prepared Spikelet layers and P25 ones. Details on the IDE geometry and the fabrication of the sandwich support-TiO_2_ layer-IDE are given in Figure S1 in Supporting Information. IDE makes possible the measurements of electrical characteristics in a two-point arrangement.

The three types of primary data from DC measurements carried out in the present study are as follows:

(i) the total current responses I_Σ_ [A] vs. time t [s] of examined layer to a sequence of irradiation pulses (optical power density 2 mW·cm^−2^, irradiation duration t_d_ = 10 s); the pulses are separated from each other by a dark period of duration t_d_ = 10 s and the voltage U applied between measuring points is 500 mV. The responses are presented as I_Σ_ in semilogarithmic scale vs. time t.
(ii) volt-ampere characteristics I_d_ [A] vs. U [V] are measured in the absence of irradiation (dark experiment); the range of applied voltage U was between −500 mV and +500 mV and it was changed in 100-mV increments; these volt-ampere characteristics are straight lines and their slopes I_d_/U represent the material conductivity σ_d_ [S] of the layer.
(iii) volt-ampere characteristics I_Σ_ [A] vs. U [V] are measured upon steady irradiation (optical power density 2 mW·cm^−2^) using the same voltage sequence as that in item (ii); the slope of the straight lines provides the total conductivity σ_Σ_ [S] of the layer.

All the DC measurements were performed using the Kiethley 4200 SCS parameter analyzer in RPM configuration. The device realized the necessary input voltage signals and collected the corresponding values of the current.

Photoactivation of the tested layers was performed in a series of experiments using different light-emitting diodes (LED) purchased from Thorlabs Inc. The wave lengths selected for photoactivation were 370, 405 and 465 nm and the optical power density was fixed to 2 mW·cm^−2^ for all the experiments. The electrical characteristics of the layers were measured (i) in dark and (ii) in photoactivation. All the measurements were performed in gas flow. The prepared layers were then placed in a PTFE measurement chamber connected to pressurized gas management, allowing a step change in the gas composition. The flow of gases was regulated through mass flow meters (Bronkhorst Ltd., Newmarket Suffolk, UK) set to 150 cm^3^·min^−1^ for all measurements. Nitrogen (N_2_) was chosen as the reference and used for electrical parameter measurements of TiO_2_ layers in the absence of photocatalytic reactions.

### 2.3. Photocatalysis in Water and Air

Regarding the photocatalysis in the aqueous phase, before the photocatalytic experiments, the photocatalyst samples were cleaned overnight by a UV light with a dominant wavelength of 365 nm and an irradiation intensity of 2.0 mW·cm^−2^ to decompose any residual organic matter on them. The aqueous solution of 4-chlorophenol (10^−4^ mol·L^−1^, which corresponds to the initial concentration c_0_ = 10 mgC·L^−1^) was photocatalytically degraded at 25 °C using photocatalytic layers (active area of 4 cm^2^ for Spikelet and P25 reference) in a 25-mL quartz cell. As the top of the liquid in the cell was open to air and the solution was intensively stirred (400 rpm), the concentration of dissolved oxygen was constant during the experiment. A Sylvania Lynx-S 11 W BLB lamp irradiated the aqueous dispersion with UV light (365 nm) at a low-power density of 1.0 mW·cm^−2^. Prior to the photocatalytic experiments, the dissolved organics were equilibrated with the photocatalyst surface for 3 h. Afterwards, for each photocatalytic experiment, aliquots, each of 100 µL, were collected from the solution in the reaction cell every 20 min for the first hour and once every hour for the next 4 h. The obtained kinetic curves were analyzed using the 1st order reaction model, the rate constants being calculated by a non-linear curve fitting using the Levenberg–Marquardt algorithm. A TOC (Total Organic Carbon) analysis was performed with a Shimadzu TOC-L Analyzer, using 20 mL of centrifuged solution. The degree of mineralization expressed as TIC (Total Inorganic Carbon) was calculated according to *TIC = c*_0_ *− TOC*.

Concerning the photocatalytic abatement of air pollutants, the photocatalytic experiment was carried out using NO or NO_2_ gases as model pollutants in a concentration of 1 ppmv. This concentration was prepared by diluting a gas mixture (50 ppmv of NO or NO_2_ in N_2_) using mass flow controllers (Bronkhorst, Ruurlo, The Netherlands) and synthetic air as the carrier gas (total gas flow ~3 L/min). A relative humidity of 50% was achieved by saturation of one half of the carrier-gas flow with water vapor. The streaming gas was then passed through the laminar flow reactor with the tested photoactive samples (50 cm^2^ of geometric area). To calibrate analyzers, the gas stream bypassed the reactor. During the photocatalytic experiments, the samples were irradiated by black light fluorescent lamps (BLB 15 W, Philips, Amsterdam, The Netherlands) emitting a dominant wavelength of 365 nm. The distance between the lamps and the coatings was adjusted to achieve an irradiation intensity of 1.0 mW·cm^−2^. This intensity corresponds to the of UV sunlight. The NO/NO_x_ concentration was monitored by a commercial NO_x_ analyzer (APNA-370, Horiba, Kyoto, Japan). The concentration of HONO was measured by custom-made analyzers based on chemiluminescence [19].

## 3. Results and Discussion

### 3.1. Structural Properties of Layers Determined by XRD and Raman Spectroscopy

#### XRD Spectra

**Figure 2:**
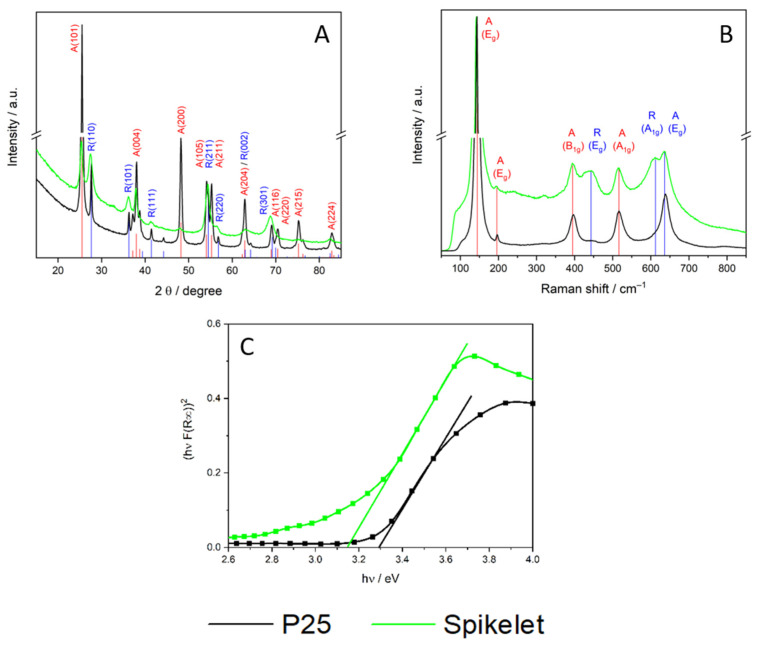
(**A**) Raman spectra, (**B**) XRD pattern and Tauc plot (**C**) of TiO_2_ Spikelet layer (green line) and P25 (black).

Spikelet TiO_2_ layers exhibit wider peaks than P25, indicating smaller particles and lower crystallinity than P25 ones (Figure 2A). In the Bragg-Brentano geometry, the molar ratio of anatase/rutile was determined as 44:56. The diffraction pattern of P25 shows reflections of both the anatase and rutile phases, indexed at (101) 25.40°, (103) 36.98°, (004) 37.90°, (112) 38.63°, (200) 48.16°, (105) 53.95° and (211) 55.16°, corresponding to a tetragonal anatase structure (space group I41/amd). Those indexed at (110) 27.31°, (101) 35.93°, (200) 39.02°, (111) 41.18°, (210) 43.98°, (211) 54.21°, and (220) 56.53° belong to a tetragonal rutile structure (space group P42/mnm). The intensity ratio of the main diffraction peaks, (anatase 101 and rutile 110) was approximately 80:20. The molar ratio anatase/rutile was then estimated to be 84:16 which is in good agreement with that found by Ohtani et al. [20].

#### Raman Spectra

Raman spectroscopy was used to verify the XRD data. The anatase TiO_2_ is represented by six Raman active modes (Figure 2B): A_1g_ + 2B_1g_ + 3E_g_ centered at: 142 cm^−1^ (E_g_), 195 cm^−1^ (E_g_), 395 cm^−1^ (B_1g_), 515 cm^−1^ (A_1g_ + B_1g_) and 636 cm^−1^ (E_g_), confirming the high phase purity of the prepared layers. The presence of the rutile phase is shown by a peak centered at 444 cm^−1^ (Eg) and 611 cm^−1^ (A_1g_). The P25 layer spectrum was dominated by the emission peaks of anatase.

#### UV-Vis Spectra

Figure 2C shows that that the transformed ultraviolet-visible spectra differed significantly. First, the band gap energy of Spikelet layer is lower (3.15 eV) than that of P25 (3.30 eV). Further, for irradiation with energies <3.2 eV, P25 did not adsorb, while for Spikelet, substantial adsorption was observed. The reasonable explanation is the different hierarchical structure and the higher concentration of rutile within the spikelet structure.

### 3.2. Morphology of Layers

#### Sorption Experiments

**Figure 3:**
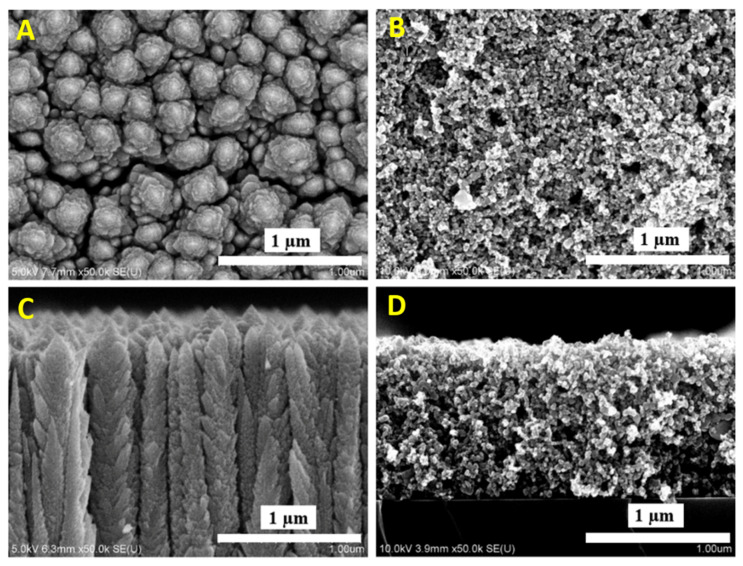
SEM micrographs of TiO_2_ layers. (**A**,**C**)—top view and cross-section of TiO_2_ Spikelet layer; (**B**,**D**)—top view and cross-section of TiO_2_ P25 reference layer, respectively.

The surface area of P25 and Spikelet TiO_2_ layers differed considerably and were equal to 50 and 120 m^2^·g^−1^, respectively. The obtained area for P25 layer is in an agreement with that for P25 powder, which shows the high degree of accessibility of the layer surface for pollutant molecules. Concerning the Spikelet layer, the large surface area is due to its unique morphology (Figure 3).

#### SEM

Figure 3 shows the comparison between the morphology of layers P25 and TiO_2_ prepared by plasma jets. The TiO_2_ loading for both types of layers is comparable, namely 2 mg·cm^−2^. Because of the different mass density of the layers, their thickness differed.

The thickness of the hierarchical layer is 7 µm, without noticeable deviation. The unique appearance of TiO_2_ crystals in the layer cross-section gives the impression of cereal Spikelets growing upward from the support. The layers on FTO-glass exhibit the same features. Anatase and rutile components cannot be distinguished from each other by view.

As the thickness of the P25 layer prepared by spin-coating was lower, achieving only about 2 µm, it was more compact for the same loading.

#### TEM and SAED

**Figure 4:**
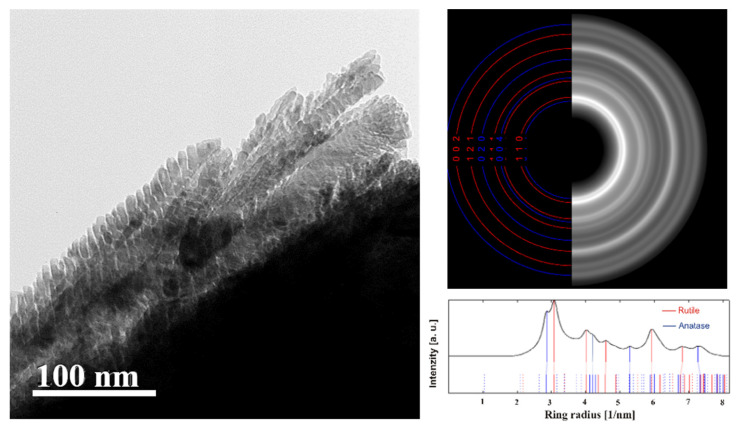
TEM micrograph of TiO_2_ nanocrystals deposited onto the TEM grid directly by plasma jet (**left**). SAED pattern (**right**); blue and red circles indicate anatase and rutile, respectively.

Even nanocrystals observed by a transmission electron microscope (TEM) exhibited both anatase and rutile components, as proven by selected area electron diffraction (SAED) (see Figure 4, right). A similar conclusion follows from details of the specific morphology of the Spikelet layer observed on a small fragment of the material using TEM, cf. Figure 4, left side. Even use of this technique did not distinguish anatase and rutile components (nanocrystals) from each other on the image by view, despite the presence of both phases being evidenced in the Spikelets by X-ray and by selected area electron diffraction (SAED) (see Figure 4, right).

#### DCDF TEM

**Figure 5:**
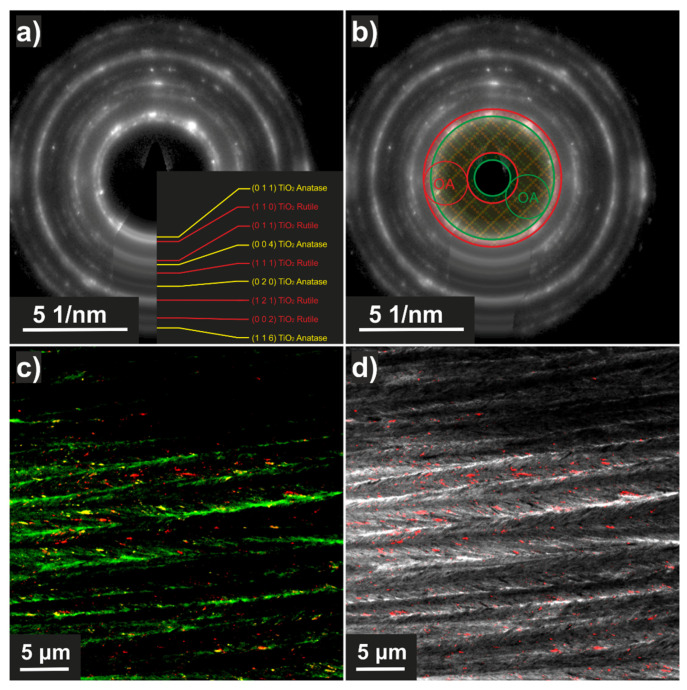
(**a**) SAED containing the two TiO_2_ phases (yellow—anatase, red—rutile); (**b**) DCDF settings (red annulus—planes (011) of anatase and planes (110) of rutile; green annulus—planes (011) of anatase (OA shows the size and position of objective aperture); (**c**) RGB composite image, in which green represents BF TEM, yellow shows the planes (011) of anatase, and red shows the planes (110) of rutile; (**d**) the composite image of grayscale BF TEM and red DCDF corresponding to planes (110) of rutile.

This technique was applied to provide information on the spatial distribution of phases for Spikelets. TiO_2_ on the Si wafer forms a columnar microstructure. The individual columns (with diameter ~200–400 nm) are separated from each other and have their own internal microstructure, similar to a spike (“twigs” emanate from the center of the columns). The “twigs” form an angle with the axis of the column in the range ~10–30°. The individual spikes contain grains of both TiO_2_ phases, anatase and rutile (Figure 5c). An analysis of the distribution of anatase and rutile in the sample was performed using the DCDF TEM technique. Due to the very close inter-planar distances of anatase (011) and rutile (110), there are considerable overlaps in the signal from both phases (Figure 5a) and moreover, there are overlaps with the bright field (BF) signal due to the size of the objective aperture (Figure 5b). However, by using specific settings (see Methods for details), it is possible to observe the signal distribution in the sample corresponding to the individual phases (Figure 5c,d): rutile (red), anatase (yellow), and undifferentiated BF TEM regions formed by both TiO_2_ phases (green). The rutile particles appear to be preferentially localized in the area of the “twigs” while anatase is distributed mainly at the edges of the formed columns.

### 3.3. DC Measurements

**Figure 6:**
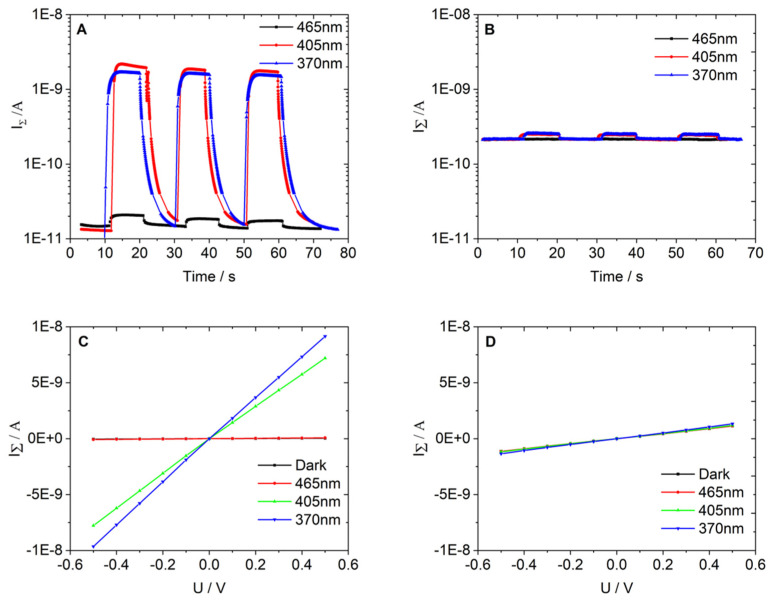
Semi-logarithmic plot of the transient photocurrent responses of Spikelet (**A**) and P25 (**B**) layers under periodic illumination by 465, 405 and 370 nm with 2 mW·cm^−2^ intensity LED. (**C**,**D**) I_Σ_-U characteristics Spikelet and P25 films in dark and illuminated conditions.

The primary data obtained from the above DC measurements on TiO_2_ layers are summarized in Figure 6 and together with additional characteristics of the systems, in Table 1 and Table 2 as well.

The sort (i) of the data is exemplified in Figure 6A for Spikelets and in Figure 6B for P25. The parameter of the response’s family obtained upon irradiation is the wavelength λ of the irradiation source.

The (ii) and the (iii) sort of the data are presented in Figure 6C for Spikelets and in Figure 6D for P25.

The generation of photocurrent upon irradiation is indicated in Figure 6A,B by a difference between the value of I_Σ_ and I_d_. This difference is denoted as I_Ph_ = I_Σ_ − I_d_ and is considered as a measure of the photocurrent I_Ph_.

A significant increase of I_Σ_ on irradiation was observed for the Spikelet layer using wavelengths of 370 and 405 nm. A much smaller contribution to I_Σ_ due to photocurrent occurred with wavelength of 465 nm. In the case of the P25 layer, we found a measurable increase of current I_Σ_ upon radiation only for wavelengths 370 and 405 nm, cf. the data in Table 1 and Table 2. This result is consistent with XRD and Raman spectroscopy data, cf. the above section on characterization. Such behavior was expected in view of the fact that the anatase does not absorb the wavelength of 465 nm. For Spikelets, the weak photocurrent for the wavelength 465 nm is the consequence of the rutile content, while for 370 and 405 nm, much higher photocurrents were observed.

The first three columns in Table 1 and Table 2 characterize the source of radiation: E is the excitation energy (eV), Ph is the photon emission rate expressed as the number of photons emitted from the radiating source per second (s^−1^). The photocurrent I_Ph_ (A) was evaluated from I_d_, I_Σ_ (A) as suggested above. The quantity e^−^ (s^−1^) represents the steady-state photoelectron generation rate expressed as a number of photoelectrons generated upon radiation per second. This quantity is related to the current I_Ph_ by the following equation:

e^−^ (s^−1^) = I_Ph_/q_e_ (1)

where charge q_e_ = 1.602 × 10^−19^ C.

The symbol EQE stays for external quantum efficiency which is defined as

EQE = e^−^/Ph (2)

It should be noted that the values of the parameter EQE were found as a rule by about 1.5 order of magnitude higher for Spikelet layers as compared with P25 ones. Specifically, (EQE)_SPIKELET_/(EQE)_P25_ is 57.6 for λ = 405 nm and 37.8 for λ = 370 nm. This is, however, not the case for the wavelength λ = 465 nm. Only in this particular case of low excitation energy, we found an opposite effect, i.e., (EQE)_Spikelet_/(EQE)_P25_ < 1, specifically, 0.245. The considerably high values of the parameter EQE for the Spikelet layers represent further characteristic feature, which makes this material unique and deserving of attention, for instance, in photocatalysis.

Primary DC data of the sort (ii) and (iii) also provide important electrical parameters of the photocatalytic layers, specifically material conductivity σ_d_ (S) (obtained from a dark experiment) and the total conductivity σ_Σ_ (S) (measured on steady irradiation) (cf. Figure 6C,D). The conductivity values σ_d_, σ_Σ_ were evaluated from the slopes of linear volt ampere characteristics treated by linear regression:

(3) $$\sigma_{i}=\frac{I_{i}}{U}$$

where i = d, Σ.

The above quantities are summarized in Table 1 and Table 2 together with their difference which represents the intrinsic photoconductivity of the material:

σ_Ph_ = σ_Σ_ − σ_d_ (4)

The description of the interphase charge transfer of the P25 and Spikelet layers can be divided into two parts: (a) in the UV region (λ < 400 nm) and (b) in the visible spectrum region (λ > 400 nm). For the ultraviolet region, the free charge carrier’s photoexcitation probability is the same in both phases (anatase and rutile). The photoexcited electrons in the anatase conduction band then diffuse to favorable electron trapping sites in the rutile since its conduction band lies approximately 0.2 eV below the edge of that in anatase [20]. On the other hand, in the visible range, the rutile phase is more activated and the reverse process takes place, i.e., the excited photoelectrons diffuse from the rutile conduction band to favorable trap states in the anatase conduction band, preventing their subsequent recombination.

Our explanation is confirmed by the work of Hurum et al. [21] and Bickley et al. [22]. The distinct band alignment and the presence of defects in the mixed-phase with a reduced gap tend to break the Ti-O bonds in the anatase and subsequently form new Ti-O bonds in the rutile. In this process, oxygen defects are generated, which increases the non-radiative deexcitation of the charge carriers that prevent their recombination [23]. Thus, it can be stated that both the free oxygen vacancies and the effective interface of the anatase/rutile phases can play a significant role in photocatalysis.

The dependence of photocurrent on the wavelength of the incident light (Figure 6A) correlates with the transformed adsorption spectra in Figure 2C. For the Spikelet layers, high photocurrents due to 370 nm and 405 nm incident light were observed, which is in accordance with the Spikelet adsorption within this range. On the other hand, for P25, the blue shift of absorption edge caused a very low level of photocurrent generated by light with a wavelength higher than 370 nm.

### 3.4. Photocatalytic Abatement of NOx and Chlorophenol

**Figure 7:**
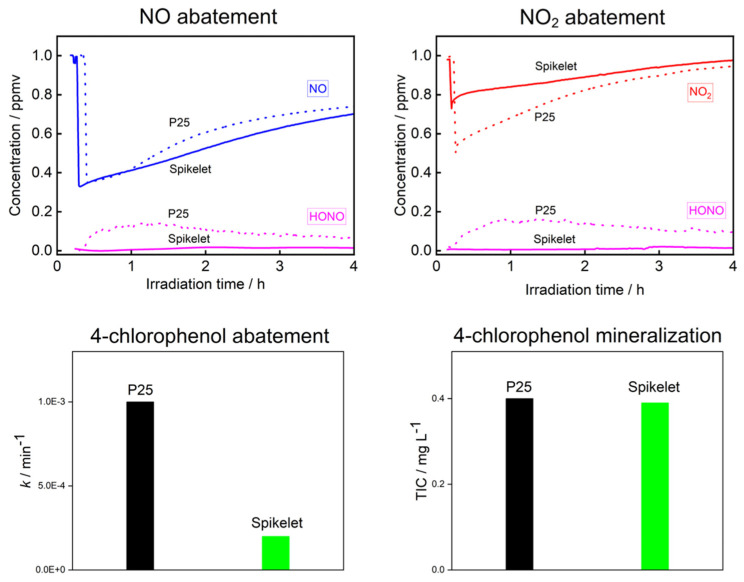
Photocatalytic removal of 1 ppm NO and 1 ppm NO_2_ on P25 and Spikelet TiO_2_ layers. Photocatalytic abatement of 4-chlorophenol and its mineralization expressed as TIC (Total Inorganic Carbon) on P25 and TiO_2_ spikelet layers.

The comparison of results from the P25 layer prepared by spin coating and the TiO_2_ layer prepared by the plasma system is shown in Figure 7. In the air flow experiments, the performance towards degradation of NO or NO_2_ was determined. For NO, the activity of both layers was comparable in the whole-time range; the selectivity, however, differed substantially. The formation of HONO was suppressed on Spikelets, while for P25, considerable concentrations of this dangerous acid were produced. Concerning NO_2_, the P25 layer was more active at the early stage of the reaction; later, after 4 h, the activity was similar. Regarding the selectivity, the same trend as for NO abatement was observed, the concentration of HONO produced being relatively high for P25 and negligible for Spikelets.

The general mechanism of NO or NO_2_ degradation is based on their oxidation on the surface of the photocatalyst due to the direct attack of the holes or OH radicals. The final product of the oxidation is nitric acid or nitrates deposited on the photocatalyst surface; however, the undesirable intermediate products NO_2_ and HONO can be released into the environment [24].

In aqueous experiments, the results were different compared to the air. P25 exhibited more than three times higher activity than Spikelets. However, what is more important is that the degree of mineralization of toxic 4-chlorophenol was practically the same for both layers. Consequently, the products of the 4-chlorophenol degradation formed on P25 were mostly organic and potentially toxic. Typically, we reported the formation of carcinogenic quinones [25]. We studied the mechanism of photocatalytic degradation of 4-chlorophenol in our previous research by employing DFT calculations based on the attack of OH radicals, leading in parallel to the hydroxylation of the aromatic ring and its opening releasing hydroperoxyl radical and hydroxyl radical, respectively. The restored OH radical can either further oxidize the primary ring opening product or attack another molecule of 4-chlorophenol [25,26,27].

From the above-mentioned results, a more general conclusion follows—on Spikelets, the formation of products in a higher oxidation state is preferable, namely that of nitric acid or nitrates in the degradation of NO or NO_2_; and, CO_2_ and H_2_O production in 4-chlorophenol degradation, respectively.

A possible explanation for the specific selectivity can be the different surface and sorption properties of both samples. Spikelets exhibit a substantially larger BET surface area than P25. Further, the percentage ratio of the anatase and rutile phases differs and the mutual interdispersion of both phases in these samples is also expected to differ. Due to their hierarchical morphology, the Spikelets can be expected to contain a higher concentration of surface defects, which can serve as traps of reactants. It was reported that these traps can favor photo-assisted adsorption, which is important for the achievement of the total oxidation (i.e., complete mineralization) of organic compounds [28]. This feature is a probable reason for the difference in selectivity achieved in the degradation of pollutants in both gaseous and aqueous phases.

## 4. Conclusions

Owing to the excellent properties, hierarchically structured layers prepared by plasma jet deposition are very promising for broad range of applications. The scale-up is easily feasible and their deposition can be realized on a variety of type substrates (metals, non-metals and/or insulants). Specifically, in the environmental photocatalysis, the hierarchical layers exhibit a unique selectivity characterized by the suppression of the formation of unwanted harmful intermediate products. In general, this type of selectivity is challenging in photocatalytic abatement of both gaseous and aqueous pollutants and very difficult to achieve. For instance, the reference TiO_2_ P25 did not exhibit this specific selectivity type in either media.

## Figures and Tables

**Table 1:** Results of DC electrical measurements for TiO_2_ Spikelet layer.

λ/nm	E/eV	Ph/s^−1^	σ_Ph_/S	σ_d_ /S, σ_Σ_/S	I_d,_ I_Σ_/A	I_Ph_/A	e^−^/s^−1^	EQE
Dark	-	-	0	2.6E−11	1.3E−11	-	-	-
465	2.66	4.7E+16	1.4E−11	4.1E−11	2.0E−11	0.7E−11	4.6E+07	9.8E−10
405	3.06	2.0E+16	4.2E−09	4.5E−09	2.2E−09	2.2E−09	1.4E+10	6.8E−07
370	3.30	4.7E+15	3.3E−09	3.3E−09	1.7E−09	1.6E−09	1.0E+10	2.2E−06

**Table 2:** Results of DC electrical measurements for TiO_2_ P25 layer.

λ/nm	E/eV	Ph/s^−1^	σ_Ph_/S	σ_d_/S, σ_Σ_/S	I_d,_ I_Σ_/A	I_Ph_/A	e^−^/s^−1^	EQE
Dark	-	-	0	4.3E−10	2.1E−10	-	-	-
465	2.66	4.7E+16	0.6E−11	4.3E−10	2.2E−10	3.0E−11	1.9E+08	4.0E−09
405	3.06	2.0E+16	0.8E−11	5.0E−10	2.5E−10	3.8E−11	2.4E+08	1.2E−08
370	3.30	4.7E+15	0.9E−11	5.1E−10	2.6E−10	4.3E−11	2.7E+08	5.3E−08

## Data Availability

The data are saved on local discs of both organisations.

## Supplementary Materials

The following are available online at https://www.mdpi.com/article/10.3390/nano11123254/s1, Figure S1: Schematic representation of IDE geometry; *W*, *S* and *L* denotes the width of the electrode fingers, their separation and length of the fingers, respectively.
