# Supplementary material for: Hierarchical TiO2 Layers Prepared by Plasma Jets
Source: Nanomaterials (Basel). 2021 Nov 30;11(12):3254. doi: 10.3390/nano11123254 (PMC8706491; doi:10.3390/nano11123254)
Supplement: Supplementary file 1 [file nanomaterials-11-03254-s001.zip › nanomaterials-1459123-supplementary.pdf]

Hierarchical TiO_2 Layers Prepared by Plasma Jets

Radek Zouzelka [1,*], Jiri Olejnicek [2], Petra Ksirova [2], Zdenek Hubicka [2], Jan Duchon [2], Ivana Martiniakova [1], Barbora Muzikova [1], Martin Mergl [1], Martin Kalbac [1], Libor Brabec [1], Milan Kocirik [1], Monika Remzova [1], Eva Vaneckova [1] and Jiri Rathousky [1,*]

[1] J. Heyrovsky Institute of Physical Chemistry, Czech Academy of Sciences, Dolejskova 3, 182 23 Prague, Czech Republic; ivana.martiniakova@jh-inst.cas.cz (I.M.); barbora.muzikova@jh-inst.cas.cz (B.M.); martin.mergl@jh-inst.cas.cz (M.M.); martin.kalbac@jh-inst.cas.cz (M.Ka.); libor.brabec@jh-inst.cas.cz (L.B.); milan.kocirik@jh-inst.cas.cz (M.Ko.); monika.remzova@jh-inst.cas.cz (M.R.); eva.vaneckova@jh-inst.cas.cz (E.V.)

[2] Institute of Physics, Czech Academy of Sciences, Na Slovance 2, 182 21 Prague, Czech Republic; olejn@fzu.cz (J.O.); pnovotna@fzu.cz (P.K.); hubicka@fzu.cz (Z.H.); duchon@fzu.cz (J.D.)

* Correspondence: radek.zouzelka@jh-inst.cas.cz (R.Z.); jiri.rathousky@jh-inst.cas.cz (J.R.); +420-266-05-34-04 (R.Z.); Tel.: +420-266-05-30-95 (J.R.)

Geometry of interdigital electrodes (IDE) to DC measurements of TiO_2 layers

An interdigital electrode (IDE) system was used to collect electric characteristics of the TiO_2 films. TiO_2 film-IDE were fabricated by magnetron sputtering using shadow masking approach. The resulting device for measurements of electric characteristics is represented by a sandwich arrangement composed of support-TiO_2 film-IDE. IDE is attached to the TiO_2 film by the above fabrication procedure. A schematic representation of IDE geometry is shown in Figure S1.

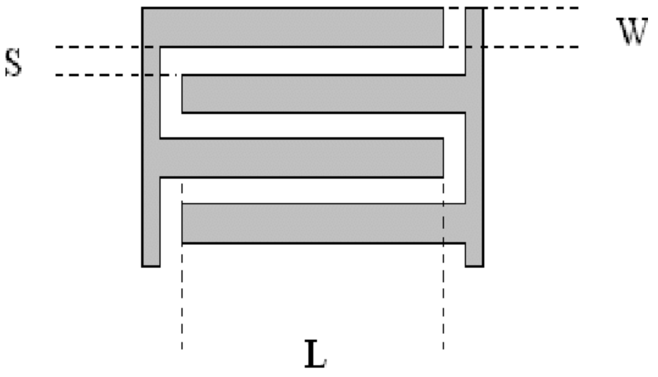

Figure S1. Schematic representation of IDE geometry; *W*, *S* and *L* denotes the width of the electrode fingers, their separation and length of the fingers, respectively.

Preliminary tests of TiO2 films-IDE sandwich have shown their relatively high surface resistivity. For this reason, the symmetrical IDE system was chosen with $W = S = 20$ μm and $L = 2$ mm. Grey areas represent the electrodes prepared by magnetron sputtering of Pt on supported TiO_2 layer . The thickness d of this Pt layer is of cca 100 nm. The thickness δ of the intrinsic TiO_2 film is of about 7 μm. The total electrodes/gaps ratio is 13/12. The total area of the IDE is 4.78 mm^2. Based on conformal imaging relationships[1]. The theoretical value of the IDE capacity was determined to be of $8.12 \cdot 10^{-13} F$. The Pt electrodes of IDE are contacted with Kiethley 4200 SCS parameter analyser via gold probes (1 μm tip radius).

Reference

1. I. Thayne, K. Elgaid, G. Ternent, „Devices and fabrication technology“. Chapter 2, in I.D. Robertson, S. Lucyszyn (Eds.), RFIC and MMIC Design and Technology. IET, 2001, London, UK pp. 40-41
